# Genomic alteration discordance in the paired primary-recurrent ovarian cancers: based on the comprehensive genomic profiling (CGP) analysis

**DOI:** 10.1186/s13048-024-01455-8

**Published:** 2024-06-27

**Authors:** Jiayin Dong, Jing Ni, Jiahui Chen, Xuening Wang, Luxin Ye, Xia Xu, Wenwen Guo, Xiaoxiang Chen

**Affiliations:** 1 Department of Gynecologic Oncology, Jiangsu Cancer Hospital, Jiangsu Institute of Cancer Research, The Affiliated Cancer Hospital of Nanjing Medical University, 42 # Baiziting street, Nanjing, Jiangsu 210009 People’s Republic of China; 2 Department of Chemotherapy, Jiangsu Cancer Hospital, Jiangsu Institute of Cancer Research, The Affiliated Cancer Hospital of Nanjing Medical University, 42 # Baiziting street, Nanjing, Jiangsu 210009 People’s Republic of China; 3 Department of Pathology, The Second Affiliated Hospital of Nanjing Medical University, 121 # Jiangjiayuan road, Nanjing, Jiangsu 210011 People’s Republic of China

**Keywords:** Ovarian cancer, Homologous recombination deficiency (HRD), Genomic loss of heterozygosity (gLOH), Comprehensive genomic profiling (CGP)

## Abstract

**Purpose:**

Ovarian cancer (OC) is characterized by a high recurrence rate, and homologous recombination deficiency (HRD) is an important biomarker in the clinical management of OC. We investigated the differences in clinical genomic profiles between the primary and platinum-sensitive recurrent OC (PSROC), focusing on HRD status.

**Materials and methods:**

A total of 40 formalin-fixed paraffin-embedded (FFPE) tissues of primary tumors and their first platinum-sensitive recurrence from 20 OC patients were collected, and comprehensive genomic profiling (CGP) analysis of FoundationOne^®^CDx (F1CDx) was applied to explore the genetic (dis)similarities of the primary and recurrent tumors.

**Results:**

By comparing between paired samples, we found that genomic loss of heterozygosity (gLOH) score had a high intra-patient correlation (r^2^ = 0.79) and that short variants (including TP53, BRCA1/2 and NOTCH1 mutations), tumor mutational burden (TMB) and microsatellite stability status remained stable. The frequency of (likely) pathological BRCA1/2 mutations was 30% (12/40) in all samples positively correlated with gLOH scores, but the proportion of gLOH-high status (score > 16%) was 50% (10/20) and 55% (11/20) in the primary and recurrent samples, respectively. An additional 20% (4/20) of patients needed attention, a quarter of which carried the pathological BRCA1 mutation but had a gLOH-low status (gLOH < 16%), and three-quarters had different gLOH status in primary-recurrent pairs. Furthermore, we observed the PSROC samples had higher gLOH scores (16.1 ± 9.24 vs. 19.4 ± 11.1, *p* = 0.007), more CNVs (36.1% vs. 15.1% of discordant genomic alternations), and significant enrichment of altered genes in TGF-beta signaling and Hippo signaling pathways (*p* < 0.05 for all) than their paired primaries. Lastly, mutational signature and oncodrive gene analyses showed that the computed mutational signature similarity in the primary and recurrent tumors were best matched the COSMI 3 signature (Aetiology of HRD) and had consistent candidate cancer driver genes of MSH2, NOTCH1 and MSH6.

**Conclusion:**

The high genetic concordance of the short variants remains stable along OC recurrence. However, the results reveal significantly higher gLOH scores in the recurrent setting than in paired primaries, supporting further clinically instantaneity HRD assay strategy.

**Supplementary Information:**

The online version contains supplementary material available at 10.1186/s13048-024-01455-8.

## Introduction

Ovarian cancer (OC) is a commonly diagnosed gynecologic malignancy, and it causes more deaths each year than any other gynecologic cancer and makes up about 4.5% of cancer-associated deaths in women worldwide [1, 2]. Still today, the main treatment for OC is maximal cytoreductive surgery and chemotherapy [3]. However, with in-depth research and extensive application on Poly ADP-ribose Polymerase (PARP) inhibitors in clinical practice, the treatment paradigm for OC has been changed [4–7]. The real-world studies have confirmed that PARP inhibitors significantly prolong the platinum-free interval (PFI) and improve the survival of OC patients [4–9]. Currently, the PARP inhibitor is recommended as first-line maintenance therapy for patients with newly diagnosed ovarian, fallopian tube, or peritoneal cancers that achieve complete or partial remission after platinum-based therapy, and the second-line maintenance therapy with the PARP inhibitors following a response to platinum-based therapy in patients with platinum-sensitive recurrent OC (PSROC) is a standard care option, irrespective of BRCA status. In addition, it is recommended that PARP inhibitor monotherapy is an alternative for the OC patients with the BRCA mutations.

It is known that anti-tumor mechanism of the PARP inhibitors (PARPi) is the synthetic lethality caused by blocking or inhibiting DNA single-strand break repair, and the key to the PARP inhibition-induced synthetic lethality is cooperative effect with homologous recombination deficiency (HRD) in the proliferating cells [10]. HRD status are primarily due to germline and/or somatic BRCA (BRCA1 and BRCA2) mutations, and there also are attributed to disability of other HRR gene (e.g., RAD51, ATM、PALB2、MRE11、CDK12 and FA) [10]. It has been demonstrated that the HRD status are clinically usefully predictors of sensitivity to PARPi therapy and platinum-based chemotherapy [8, 9, 11, 12], and the examination for HRD status has widely been recommended in treatment of OC, breast cancer, prostate cancer and other tumors [13, 14]. In tumor cells, HRD status can cause specific, stable genomic alterations that include short variants (SVs, include single-nucleotide variants and insertions/deletions), gene copy number variations (CNVs) and chromosomal structural abnormalities [15]. These genomic alterations as genomic signatures of HRD status (also called genomic scars) can be identified and quantified by next-generation sequencing (NGS)-based platforms only on tumor tissue [15], and the NGS-based commercial BRCA mutation test and HRD status assessment have been developed and recommended in clinical practice [16, 17].

OC tends to recur, especially the advanced-stage disease that nearly three-quarters have a recurrence within the first 2 years after initial treatment [18]. Therefore, the pressing questions facing oncologists are whether early HRD evaluation is helpful for clinical decision making in OC recurrent setting, and how their genomic signatures have changed in the recurrent or progressive setting. Previous work by Patel et al. indicated that HRD status information of primary OC can guide treatment decisions of the recurrent tumors [19]. Here, our study looking at comprehensive genomic profiling (CGP) of paired primary and recurrent OC samples found that they had a high intra-patient concordance of genetic alteration events (in especial SVs) while the recurrent tumors were still characterized by elevated gLOH score, high frequency of structural variants (CNVs and rearrangement events) and enriched altered genes of cancer stem cell (CSC)-related signaling pathways.

## Materials and methods

### Patients and tissues

Formalin-fixed paraffin-embedded (FFPE) tissue specimens of the primary tumors and their first platinum-sensitive recurrences were collected upon informed consent from OC patients who underwent a primary debulking surgery (PDS) or neoadjuvant chemotherapy followed by interval debulking surgery (NAC/IDS) and a secondary debulking surgery from 2014 to 2021. The informed consent was obtained from all patients before FFPE sample collection in accordance with the Declaration of Helsinki and under the study procedure approved by the Ethics Committee of the Affiliated Cancer Hospital of Nanjing Medical University (2022SCIENCE-003).

### Comprehensive genomic profiling (CGP) analysis

All enrolled FFPE tissue sections were pathologic reviewed to confirm sufficient tumor fraction (≥ 20% tumor cells) and they used to genomic DNA extraction. The extracted genomic DNA was analyzed by CGP analysis of FoundationOne^®^CDx (F1CDx). Sequencing methods of the CGP analysis was reported and validated previously [16]. Briefly, 50 ng genomic DNA was employed to adaptor-ligation, and followed by captured library construction for the coding exons and frequently rearranged introns of 324 cancer related genes. The captured libraries were sequenced on Illumina HiSeq platform with a mean exon coverage depth of > 500×. Resulting sequence data was analyzed using in-house developed bioinformatics analysis pipeline of Foundation Medicine Inc. (MA, USA) to determine genomic variants including SVs, CNVs, genomic rearrangements, tumor mutation burden (TMB), microsatellite instability (MSI) status and gLOH. A cutoff of 16% score was applied for the gLOH rating of OC, and the patients with an LOH score ≥ 16% were recognized as gLOH-high and less than 16% as gLOH-low. In this study, the HRD status have been evaluated based on the gLOH score and BRCA mutations, a positive HRD (HRD-positive) status can either be defined as the presence of (likely) deleterious/pathological BRCA1/2 mutations or gLOH-high [20, 21].

### Genomic aberration profiling data analysis

To explore the genomic alteration profiles and their divergence between the primary and recurrent tumors, the customized R script with publicly available packages, maftools, clusterProfiler and PathwayMapper were adopted to perform exploratory bioinformatics analysis. Mutational signature analysis was used to perform etiologic inference and the analysis pipeline in maftools was invoked to extract mutational signatures in the pairs. The etiologic (dis)similarities between the primary and recurrent tumors were interpreted by calculating the cosine similarity of the extracted mutational signatures to the COSMIC signatures, a catalog of unique combinations of mutation types that reveals the diversity of mutational processes underlying cancer development. Oncodrive genes were identified by oncodrive function of maftools, which takes advantage of mutational hot-spots of cancer genes to determine cancer drivers based on the oncodriveCLUST algorithm [22]. Cluster Profiler was used to perform KEGG pathways enrichment analysis of the altered genes. The genomic alteration profiles of the TCGA OC cohort [23] were downloaded by cBioportal and compared with the genetic variation data from the pairs. PathwayMapper was employed to map the gene mutational frequencies of the study samples and TCGA OC cohort in canonical signaling pathways.

### Statistical analysis

Unpaired t-test, paired t-test, Mann-Whitney U test, or Wilcoxon’s signed-rank test were conducted for between-group differences of continuous variables when appropriate, and Chi-squared or Fisher’s exact tests were utilized to find significant genetic alternations between the primary and recurrent tumors. In all cases, a *p*-value less than 0.05 was considered statistically significant.

## Results

### Patient characteristics

A total of paired 40 primary-recurrent FFPE tumors from 20 sporadic OC patients were included, and the patient demographics are shown in Table 1. These patients ranged from 45 to 68 years-old, with a median age of 48.0. All cases were clinically and pathologically diagnosed as OC, and there were 1 case (5.0%) of stage I, 4 cases (20.0%) of stage II, 13 cases (65.0%) of stage III, and 2 cases (10.0%) with unknow stage tumors. Among these patients, seven of them (35.0%) received a neoadjuvant carboplatin (CBP)-paclitaxel (PTX) chemotherapy followed by debulking surgery. All patients received postoperative platinum-based adjuvant chemotherapy, of whom 13 patients were treated with CBP-PTX chemotherapy, 3 with PTX plus cisplatin (DDP), and 4 with other platinum-containing regimens. In addition, there are five patients treated with Olaparib after their cytoreductive surgery. Three of the treated patients had high gLOH scores, two of whom had pathologic BRCA1 mutations (*BRCA1 c. 2687delG* and *BRCA1 c.3607 C > T*). The other two treated patients were HRD-negative. The two patients with pathologic BRCA1 mutations received PARPi second-line maintenance therapy and multi-line maintenance therapy, respectively, and the patient with high gLOH, whose primary and recurrent tumor samples were 18.6% and 21.8%, respectively, received Olaparib multi-line maintenance therapy. The two HRD-negative patients received Olaparib second-line maintenance therapy and multi-line maintenance therapy, respectively. The interval between PDS or NAC/IDS and subsequent secondary debulking surgery ranged up to 60 months, with the median interval 29.5 months. The PFI time ranged from 6.0 to 36.5 months with median of 15.0. The patients were followed for a median of 63 months (range, 34.0–108 months), during which time 7 patients (35.0%) died and 1 case lost to follow-up.

**Table 1:** Baseline characteristics of the enrolled patients in the study

Characteristics	Number (%)
Total number of the enrolled patients	20 (100)
Age at diagnosis, Median (range), years-old	48 (45–68)
FIGO Staging Classification	
I II III Unknow	1 (5.0) 4 (20.0) 13 (65.0) 2 (10.0)
Tumor histology	
Serous Others	17 (85.0) 3 (15.0)
Neoadjuvant treatment	
Yes (PTX plus CBP) No	7 (35.0) 13 (65.0)
Targeted molecular therapy (Olaparib)	
Olaparib No	5 (25.0) 15 (75.0)
First-line chemotherapy	
PTX plus CBP PTX plus DDP	13 (65.0) 3 (15.0)
Other platinum-containing regimens	4 (20.0)
Operation interval, Median (range), months	29.5 (13.5–60.0)
Platinum-free interval (PFI), Median (range), months	15.0 (6.0-36.5)
Follow-up, Median (range), months Live Death Lose	63.0 (34.0-108.0) 12 (60.0) 7 (35.0) 1 (5.0)

FIGO, International Federation of Gynecologists and Obstetricians. PTX, Paclitaxel. CBP, Carboplatin. DDP, Cisplatin/ cis-diamminedichloro-platinum

Platinum-free interval (PFI), which is calculated from the last platinum-based chemotherapy to the time of recurrence; Operation interval, refers to the interval between the PDS or NAC/IDS and the secondary debulking surgery

### Brief overview of HRD, TMB and microsatellite status in sporadic serous ovarian cancer

In total, we observed that 5 pairs had (likely) pathogenic BRCA1 mutations (3 frameshift mutations: *c.869delT*, *c.2302delA* and *c.2687delG*; 1 nonsense mutation: *c.3607 C > T*; and 1 splice-site mutation: c.441 + 1G > A), and one pair carried a BRCA2 frameshift mutation (*c.9097_9098insA*) (Table S1). One pair harbored a BRCA2 missense mutation, *p.V2109I* (*c.6325G > A*), but there was another BRCA2 missense mutation, *p.E747G* (*c. 2240 A > G*) identified in the recurrent sample (Table S1). The *p.V2109I* was more confidently predicted to be a benign/neutral genetic variant by SIFT, Polyphen, CADD, REVEL, MetaLR and PROVEAN platforms. The *p.E747G* was predicted to be “likely pathogenic” by Polyphen and PROVEAN tools, but it was considered as “likely benign (tolerated)” using SIFT, CADD, REVEL and MetaLR predictors.

**Fig. 1:**
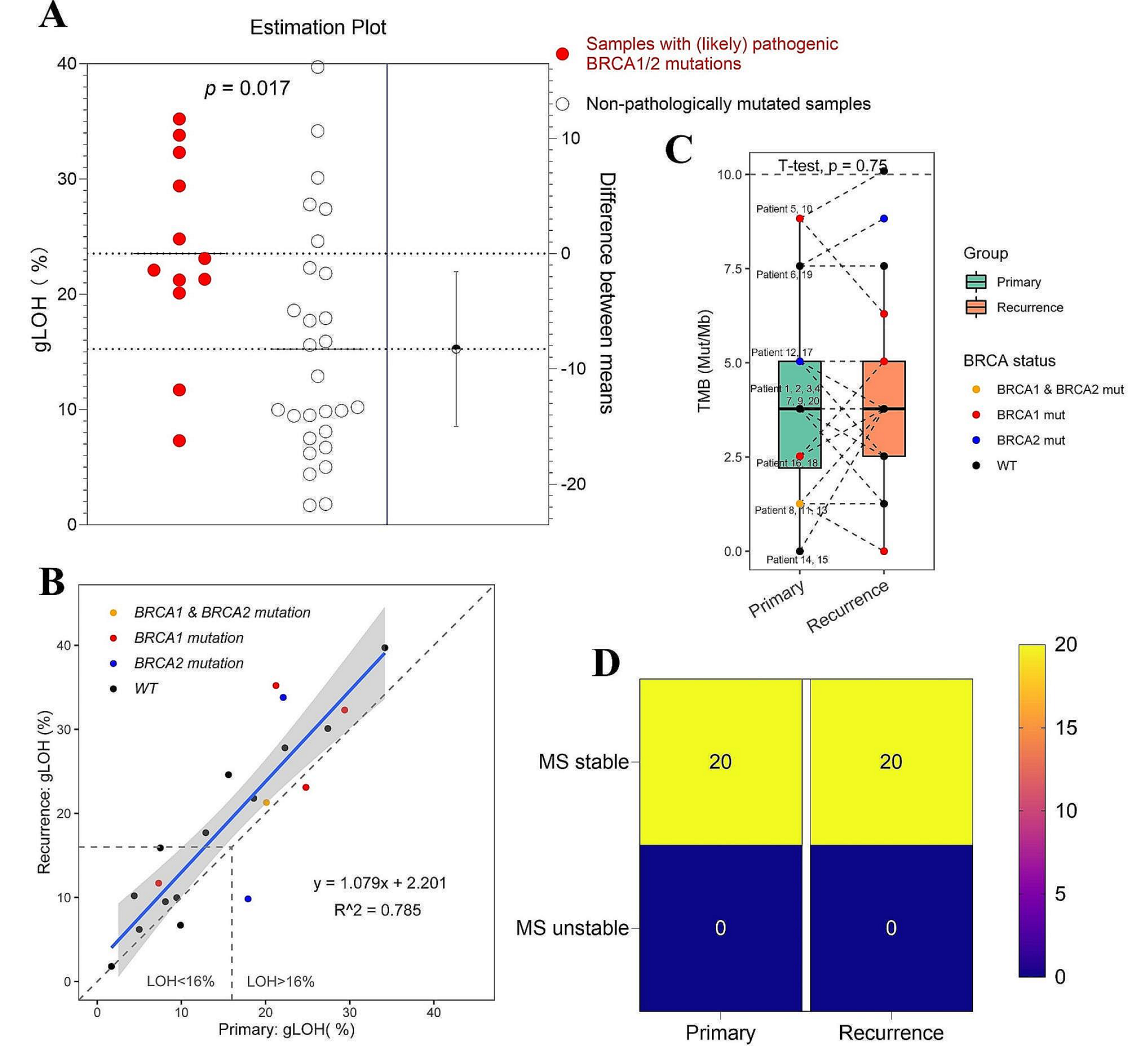
Comparison of gLOH, TMB and microsatellite stability status in the primary-recurrent OC pairs. (**A**) Estimation plot of correlation analysis between (likely) pathogenic BRCA1/2 mutations and gLOH scores. P value is calculated by two-tailed t-test. (**B**) Correlation between gLOH in primary (x) and recurrent (y) tumor samples. The grey horizontal and vertical dashed lines indicate the cut-off (16%) between HRD-positive and HRD-negative tumors for both the primary and recurrent tumors. The strength of the linear relationship between the two different variables, gLOH of the primary and recurrent tumors, is given by the correlation coefficient. BRCA1/2 mutation status is shown by the colored dots. (**C**) Comparison of TMB in primary and recurrent tumor samples. *P* value is generated from two-tailed paired t-test. BRCA1/2 mutation status is shown by the colored dots. The patient numbers are shown. (**D**) microsatellite stability status of the 20 pairs

As expected, we observed a significant positive correlation between the BRCA1/2 mutation and gLOH scores (Fig. 1A-B), with pathologically mutated samples having higher gLOH scores than non-pathologically mutated samples in all cases (23.5 ± 8.43% vs. 16.6 ± 7.02%, *p* = 0.017) (Fig. 1A), even in both primary and recurrent tumors (Fig. S1A) showing the patients with the pathogenic BRCA1/2 mutations (*n* = 6) had numerically higher gLOH score compared to those without (*n* = 14) (21.0 ± 7.40% vs. 13.6 ± 6.74%, *p* = 0.117 in the primary and 26.0 ± 9.31% vs. 19.7 ± 6.37%, *p* = 0.080 in the recurrence). Additionally, the high intra-patient correlation between the gLOH score among pairs was observed (r^2^ = 0.785, Fig. 1B, S1B). The primary tumors had a median gLOH score of 16.76% with a range from 1.69 to 34.17, of which 10 samples (50%) were gLOH-high (Fig. 1B). In the recurrent tumors, they had a median gLOH score of 18.90% with a range from 1.80 to 39.71, and 55% of these samples (11/20) were gLOH-high (Fig. 1B). In all, 80% (16/20) recurrent tumors were numerically higher than their paired primary tumors, and the other 20% (4/20) were numerically lower than their counterparts (Fig. 1B, S1B).

In the study, the median TMB of the primary tumors was not significantly different from that of the recurrent tumors (4.00 [IQR, 1.50-5.00] vs. 4.00 [IQR, 3.00–5.00]; *p* = 0.75) (Fig. 1C). As observed in the primary tumors, 55% (11/20) cases harbored HRD (pathogenic BRCA1/2 mutations or gLOH-high), and no difference in median TMB was observed between the HRD cases and non-HRD cases (3.00 [IQR, 1.00–5.00] vs. 4.0 [IQR, 4.00-6.50]; *p* = 0.259) (Fig. S2). Consistent with the observation of the primary tumors, TMB was similar in HRD and non-HRD recurrent samples (4.0 [IQR, 1.50–5.75 vs. 3.50 [IQR, 3.00–5.00]; *p* = 0.937) (Fig. S2). Additionally, it is noteworthy result that all cases, both primary and recurrent tumors, were microsatellite stable (MSS) (Fig. 1D).

### Genetic mutational events did differ significantly between the primary and recurrent tumors

**Fig. 2:**
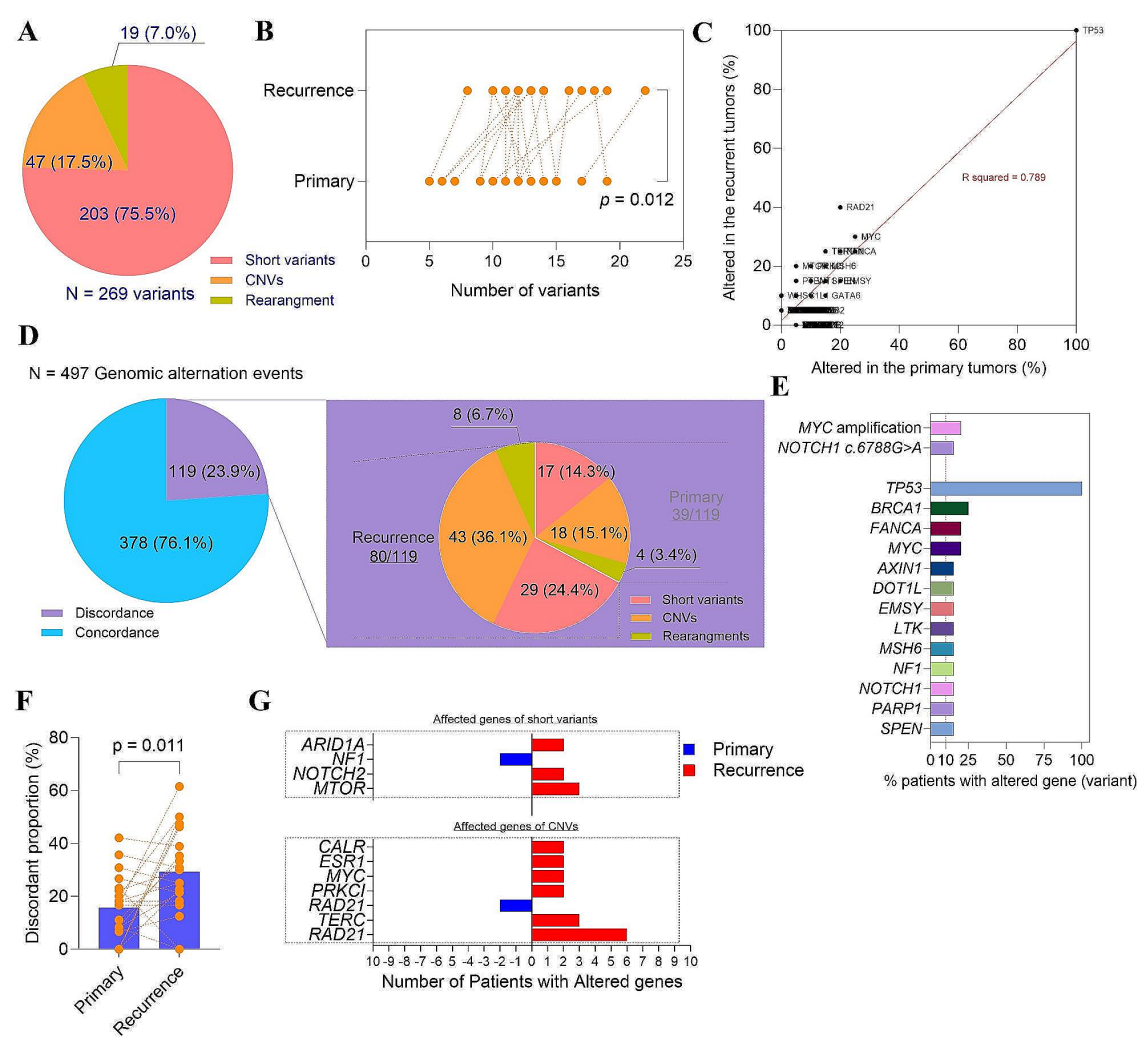
Mutational profiles differed between the primary and recurrent tumors. (**A**) Compositional types of the 269 identified variants. (**B**) Comparison of genetic alteration events between the primary and recurrent tumors. P value is computed by two-tailed paired t-test. (**C**) Correlation analysis of the genetic alteration profile between the primary and recurrent tumors. (**D**) Composition of 119 discordant genetic alteration events in primary and recurrent tumors. (**E**) Genetic variants with a frequency greater than 10% in concordant mutational events. (**F**) Comparison of the discordant proportion between the primary and recurrent tumors. P value is calculated by two-tailed paired t-test. (**G**) Frequently variants (more than 2 out of the 20 patients) of the discordant genetic alternations in the primary and recurrent tumors

A total of 497 genomic mutational events (Table S2) that involved 269 variants of 170 genes were detected in 20 primary-recurrent tumor pairs, and there were 203 (75.5%) SVs, 47 (17.5%) CNVs and 19 (7.0%) rearrangements (Fig. 2A). Comparing with the primary tumor samples, more mutational events were identified in their paired recurrences (13.5 ± 3.41 vs. 11.4 ± 3.70, *p* = 0.012) (Fig. 2B). In terms of variant type, SVs (9.60 ± 2.28 vs. 9.00 ± 2.38, *p* = 0.036), copy number variants (3.10 ± 2.47 vs. 1.85 ± 2.52, *p* = 0.077) or rearrangement events (0.75 ± 0.97 vs. 0.55 ± 0.76, *p* = 0.259) detected in the recurrences were more numerically than their primary counterparts (Fig. S3A-C). Correlation analysis showed a strong correlation of the genetic alteration profile between the primary and recurrent tumors (r^2^ = 0.789) (Fig. 2C), especially the mutational events of SVs (r^2^ = 0.889) (Fig. S3D). In contrast, the CNVs and rearrangements were weakly correlated between the two groups (r^2^ = 0.560 and 0.241, respectively) (Fig. S3E-F). Specifically, 378 events were concordant (concordance 76.1%) in the 497 mutational events and detected both in the pairs (Fig. 2D). The concordant events covered 283 variants, and the frequently variants (> 10% patients) were MYC amplification (4/20, 20%) and NOTCH1 c.6788G > A (3/20, 15%), and the altered genes with a frequency higher than 10% were TP53 (20/20, 100%), BRCA1 (5/20, 25%), FANCA (4/20, 20%), MYC (4/20, 20%), AXIN1 (3/20, 15%), DOT1L (3/20, 15%), EMSY (3/20, 15%), LTK (3/20, 15%), MSH6 (4/20, 20%), NF1 (4/20, 20%), NOTCH1 (3/20, 15%), PARP1 (3/20, 15%) and SPEN (3/20, 15%) (Fig. 2E). The remaining 119 mutational events were discordant events in either primary or recurrent tumors, and 39 (32.8%) in the primary tumors, and 80 (67.2%) in the recurrent tumors (Fig. 2D). The discordant proportion varied with the type of genomic alteration and tumor, being the highest for CNVs in recurrent tumors (36.1%, 43/119), and the lowest for rearrangements in the primary tumors (3.4%, 4/119) (Fig. 2D). The recurrent tumors had a significantly increased proportion of discordant mutational events compared to their paired primary tumors (29.3% ± 16.4% vs. 15.7% ± 12.2, *p* = 0.011) (Fig. 2F). With respect to the mutation types, the discordant fraction of SVs in the recurrent tumors was significantly higher than in their primary tumors (15.6% ± 12.1% vs. 9.74% ± 9.68%, *p* = 0.034) (Fig. S4 A), and the proportions of the CNVs and rearrangements were numerically higher than in their primary tumors (52.9% ± 41.8% vs. 21.8% ± 36.7%, *p* = 0.053 for CNVs, and 25.8% ± 38.8% vs. 12.5% ± 31.9%, *p* = 0.313 for rearrangements) (Fig. S4 B-C). In the discordant SVs, the affected genes that observed in more than 1 out of the 20 patients were NF1 (2/20, 10%) in the primary tumors, and ARIDIA (2/20, 10%), NOTCH2 (2/20, 10%) and MTOR (3/20, 15%) in the recurrent tumors (Fig. 2G). Within the discordant CNVs, the genetic variants with a frequency greater than or equal to 10% were RAD21 amplification (2/20, 10%) in the primary tumors and CALR amplification (2/20, 10%), ESR1 amplification (2/20, 10%), MYC amplification (2/20, 10%), PRKCI amplification (2/20, 10%), TERC amplification (3/20, 15%), and RAD 21 amplification (6/20, 30%) in the recurrent tumors (Fig. 2G). For the rearrangements, each rearrangement is an all-too-rare event whether in the primary or recurrent tumors (Fig. S5A), with the average number of discordant variants per sample being 0.3, and even concordant variants per sample was 0.35 on average (Fig. S5B).

### Genomic findings: sporadic ovarian cancer has an etiological link with decreased HRR capacity

**Fig. 3:**
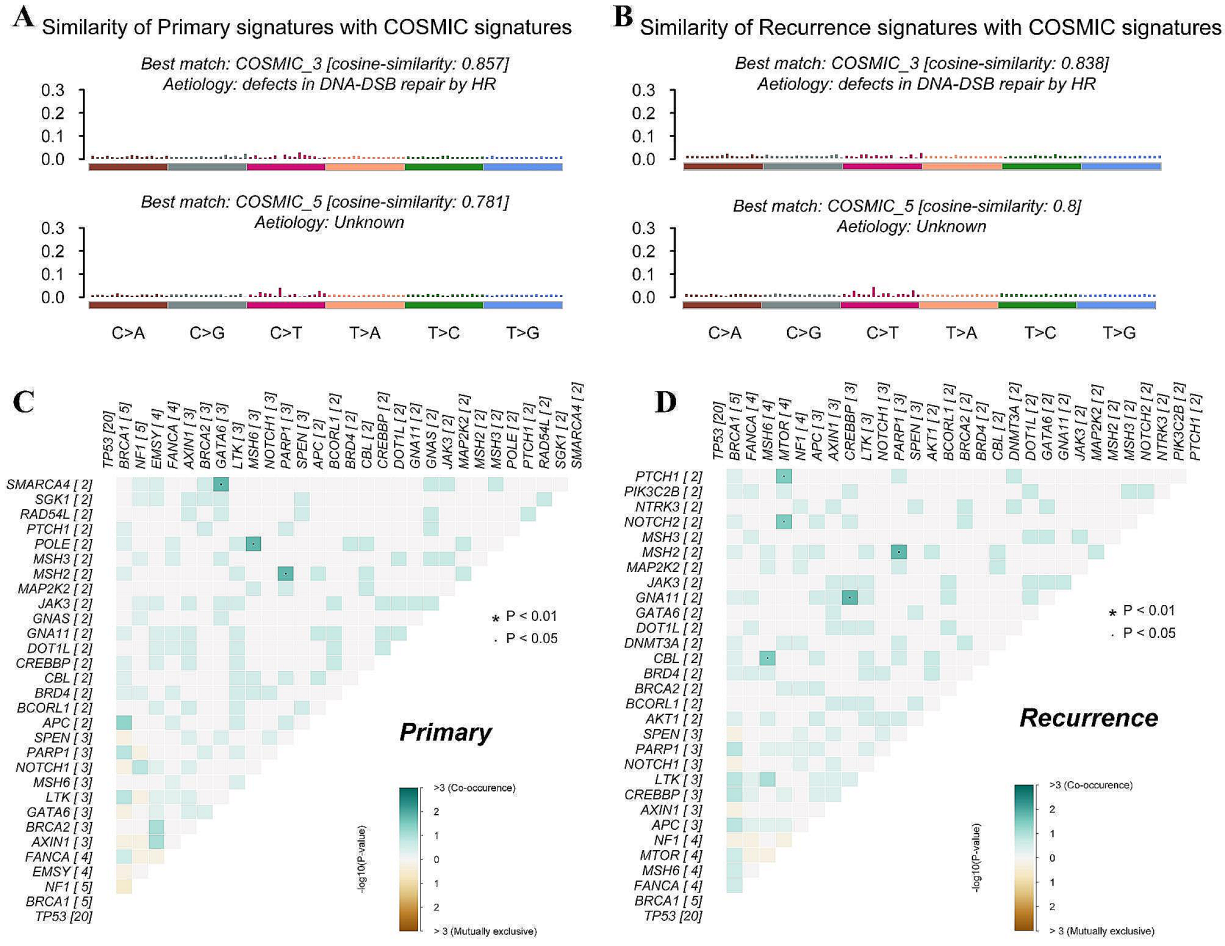
Mutational signature analysis in genetic alternation profiling of the primary and recurrent tumors. (**A**-**B**) Computed cosine similarity against COSMIC signatures and proposed aetiology for the primary (**A**) and recurrent (**B**) tumors are visible in the graphical representation. (**A**-**B**) Mutational signatures deciphered from the base substitutions identified in the genomes of 20 primary-recurrent tumor pairs. (**C**-**D**) Co-occurring and mutually exclusive somatic alternations across genes in the primary (**C**) and recurrent (**D**) tumors. P values are calculated by Chi-squared and Fisher’s exact tests

Mutational signature analysis was used for etiologic inference, and the cosine similarity of the two signatures in primary tumors to the COSMI 3 (etiology: defects in DNA-DSB repair by HR) and COSMI 5 (etiology: unknown) signatures was observed to be as high as 0.857 and 0.781, respectively (Fig. 3A). Similarly, the computed cosine similarity of the signatures in the recurrent tumors with the COSMIC signatures were 0.838 matched COSMI 3 signature and 0.800 matched COSMI 5 signature (Fig. 3B). The identified mutational signatures and the COSMI 3 signature had a similarity higher than 0.8, suggesting that they are all associated with the defects in DNA-DSB repair by HR. The relationships of the genetic alternations found in the cohort were analyzed in the primary and recurrent tumors by co-occurring and mutually exclusive alterations across genes, and the significant findings were co-occurrences of MSH2 and PARP1, SMARCA4 and GATA6, and POLE and MSH6 in the primary tumors (Fig. 3C). In the recurrent tumors, there were significant co-occurrence between MSH2 and PARP1, PTCH1 and MTOR, NOTCH2 and MTOR, GNA11 and CREBBP, CBL and MSH6 (Fig. 3D).

**Fig. 4:**
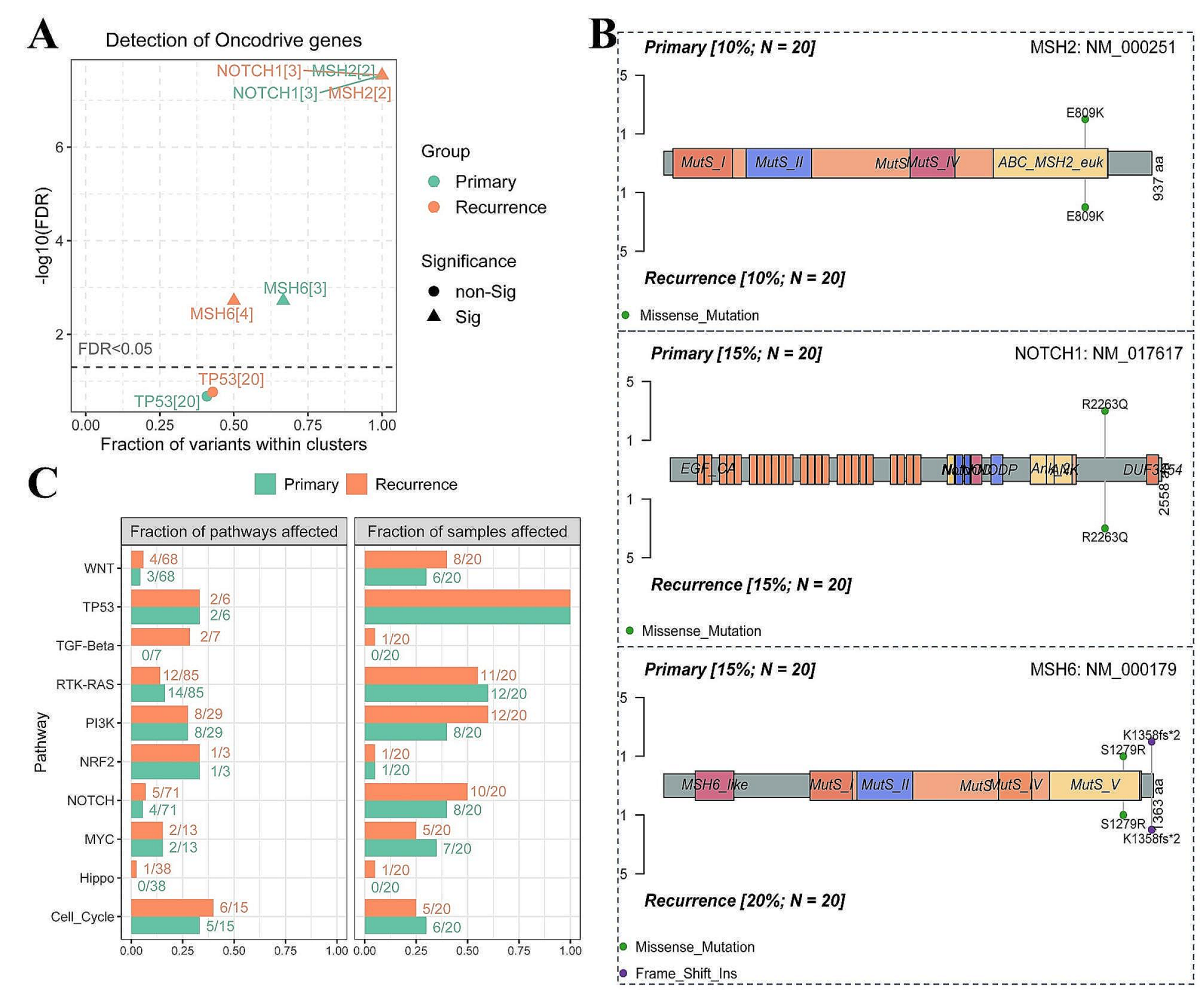
Identifying cancer driver genes and pathways among all somatic mutations detected in a cohort of the primary and recurrent tumors. (**A**) Comparison and identification of cancer drivers in the paired primary-recurrent tumors. (**B**) Mutational analysis of candidate cancer driver genes (NOTCH1, MSH2, and MSH6) in the primary and recurrent tumors. (**C**) Enrichment analysis of the mutated genes identified in genomes of the primary and recurrent tumors for 10 TCGA canonical signaling pathway

Further we have identified cancer driver genes based on all genetic alternations detected in those primary and recurrent tumors and have found three candidate cancer driver genes of MSH2, NOTCH1 and MSH6 in the primary and recurrent tumors (Fig. 4A). Among them, a MSH2 missense mutation (*p.E809K*) was found in 10% (2/20) of the pairs, and the *NOTCH1 c.6788G > A* (*p.R2263Q*) mutation was observed in 15% (3/20) of the pairs (Fig. 4B). For MSH6 alterations, 1 pair had a missense mutation of *MSH6 p.S1279R* and 2 pairs had a frameshift mutation of MSH6 p.K1358fs*2 (Fig. 4B). In addition, there was a patient with a splice site mutation, *MSH6 c.4002-3_4023 > T*, that was detected only in the recurrent tumor sample and not in the primary tumor sample (Table S2). Additionally, we analyzed 10 signaling pathways with frequent genetic alternations in the TCGA ovarian cancer cohort by Cluster Profiler and Pathway Mapper, and compared fraction of genetic alternations and proportion of sample affected by those genetic alternations in the 10 pathways between the primary and recurrent tumor sample groups. The pathways are WNT signaling, TP53-related, TGF-beta signaling, receptor-tyrosine kinase (RTK)-RAS kinase signaling, PI3Kinase signaling, NRF2 signaling, NOTCH signaling, MYC signaling, Hippo signaling and Cell cycle pathways (Fig. 4C). We observed the fraction of affected genes involved in these signaling pathways and the proportion of sample affected were similar between the two groups (primary and recurrent tumors). However, we found that gene alternations of TGF-beta signaling and Hippo signaling pathways were occurred only in recurrent tumors while these alternations were not found in the primary tumors (Fig. 4C).

## Discussion

The most common causes of HR-deficiency currently known to confer sensitivity to PARP inhibitors are loss-of-function of HRR-related genes through pathogenic mutation (germline or somatic mutations) or epigenetic inactivation in which there is a frequent event in OC [24], especially in the high-grade disease about half have aberrations of HRR genes [23]. For OC, most HR-deficiency result from BRCA1/2 pathogenic mutations [23]. In this cohort, the frequency of (likely) pathologic BRCA1/2 mutations was 30% (6/20) (Table S1).

OC is the most mortal gynecological malignant tumor and recurs at a high rate [18]. The recurrence rate of the early-stage disease is almost 25%, while the advanced stage disease can reach 80% within 1 to 2 years after initial treatment [18]. This raises an interesting topic in terms of whether HRD evaluation of the primary tumors is justifiable for the treatment of their recurrent setting. Patel et al. study showed that the BRCA1/2 mutational status and HRD scores were highly consistent between primary and their recurrent tumor samples, and they concluded HRD relevant genetic information of primary ovarian cancer can guide treatment decisions in their recurrences [19]. Likewise, comparative sequencing analysis of primary and recurrent colorectal cancer (CRC) also suggested a high degree of genetic concordance between primary and progressed staging [25]. Here, by comparing the CGP of the primary-recurrent tumor pairs of Chinese OC patients, it was confirmed that most of the mutational events (76.1%) were shared in the primary-recurrent pairs, and particularly the SVs had a strong correlation (r^2^ = 0.889), relative to the CNV (r^2^ = 0.560) and rearrangements (r^2^ = 0.241). Therefore, BRCA1/2 mutation is largely consistent across the pairs, except for one pair in which an additional BRCA2 mutation (*BRCA2 p.E747G*) was identified in the recurrent tumor. Although, a high intra-patient correlation of gLOH score was also observed among the 20 evaluable pairs (r^2^ = 0.785), there was still inconsistency in 15% of the patients (3/20) adjudicated for gLOH classification, with two-thirds patients having their gLOH scores escalated to gLOH-high in the recurrent setting and one third of patients having gLOH score downgraded to gLOH-low in their recurrence. Moreover, there was a paradox in one patient (5%) of our study cohort that the samples with pathological BRCA1 mutations had a low gLOH score, and this inconsistency could be a compensatory function of DNA repair system, or a result of functional compensation for BRCA1 heterozygote mutation. It is not clear whether the patients with pathological BCRA1/2 mutations but low HRD scores can benefit from PARP inhibitors or platinum-based drugs, which needs to be further investigated in clinical studies. Interestingly, our data showed gLOH score is significantly increased in 80% of the recurrent tumors compared to their primary counterparts (*p* = 0.007), and it may be attributed to more genomic scar accumulation during the recurrent setting. Based on binary classifier of gLOH status, 50% of the primary samples in the study cohort were gLOH-high and 55% of the recurrent samples were gLOH-high. Since PARP inhibitor drugs reveal the definite beneficial effect in treating OC, it is crucial to proactively identify these patients, especially in PSROC patients. Thus, the results support a cautiously optimistic use of genetic scar-based HRD score of the primary in treatment decision-making for PSROC patients, and it is recommended to re-evaluate the HRD status of PSROC when conditions permit (sample availability and economics permitting).

In this study, the CGP analysis revealed a high concordance of genetic alternation events in the 20 pairs, and the genomic concordance varied according to the variant types, with the highest concordance being observed for SVs, followed by CNVs and rearrangements. Of these concordant genetic alterations, two variants of MYC amplification (20%) and NOTCH1 c.6788G > A (15%) are more prevalent in the OC cohort. It has been shown that NOTCH1 and MYC involve oncogenic NOTCH signaling pathways [26], in which NOTCH signaling activation is dependent on MYC upregulation, a NOTCH1-MYC regulatory route that is an attractive target for the treatment of T-cell acute lymphoblastic leukemia (T-ALL) [26, 27]. However, the biological role and clinical significance of NOTCH1-MYC pathway in OC deserve to be investigated in further in vivo and large-scale-patient studies. Additionally in the concordant SVs we observed that the frequently altered genes were enriched in DNA repair pathway and especially in the HRR pathway, which is similar to the results reported by Angeliki et al [28]. This genetic concordance is indirectly verified by mutational signature and oncodriver analyses, and indicated that the primary and recurrent tumors have similar etiological factor (Aetiology: defects in DNA-DSB repair by HR) and candidate cancer driver genes (MSH2, NOTCH1 and MSH6). Even though a high concordance of genetic alternation events between the pairs, a genetic variability still existed. Beside the finding that more genetic mutational events were detected in the recurrent tumors compared to their counterparts, whether SVs, CNVs or rearrangements, it was observed that rearrangements among pairs were rare heterogeneous genomic alternation events, while the recurrences had more discordant CNVs. Therefore, this paradox of high genetically similarity and variability that coexist supports the genetic continuity between primary-recurrent OC and suggests that the tumorigenesis may derive from genomic SV events and that discordant genomic alternations (CNVs and rearrangements) may confer behavioral phenotypic variability of recurrent OC that differ from the primary.

It is notable that recurrent samples had the highest proportion of genetically discordant events (67.2% of total discordant events, 80/119), the majority of which were CNVs (53.8%, 43/80). These CNVs were predominantly composed of gene amplifications (93%, 40/43), and implied that recurrent OC are more prone to gene amplification. Among these CNVs, CALR amplification (10%), ESR1 amplification (10%), MYC amplification (10%), PRKCI amplification (10%), TERC amplification (15%) and RAD 21 amplification (30%) were frequent CNVs in the recurrent OC. Evidently, most of these CNVs are involved in oncogene signaling [29], which is associated with aggressive behavior and poor prognosis in cancer [29]. One of them, RAD21 is noteworthy as a frequently amplified oncogene in the cohort (20% of the primary and 40% of the recurrence). Biologically, RAD21 is directly involved in genome organization as a core component of cohesin complex [30]. Deng et al. reported that RAD21 amplification is associated with suppression of interferon (IFN) signaling pathway, which controls T cell activation and promotes immune escape of OC [31]. With this result, RAD21 amplification is a potential biomarker for immune checkpoint inhibitor therapy (PD-1 antitumor therapy) in OC [31]. A case report of Sabbatino et al. demonstrated that RAD21 amplification in metastatic intrahepatic cholangiocarcinoma (ICC) is related to clinical benefit of Olaparib (PARP inhibitor) treatment [32], suggesting that it may be a predictive biomarker of PARP inhibitor efficacy in ICC. Therefore, further clinical observations of RAD21 amplification in immunotherapy and PARP-targeted therapy for solid tumors are warranted. Furthermore, the results of ClusterProfiler and PathwayMapper analyses integrating the TCGA OC cohort showed that the altered genes in the PSROC were significantly enriched in the TGF-beta and Hippo signaling pathways. Numerous studies have illustrated that these two signaling pathways play important roles in stem cell renewal and stemness maintenance [33, 34]. It could be speculated that the enrichment of these relevant gene alternations may be related to the tumor stem cell clones within OC residuals. Finally, the rearrangement event is an all-too-rare event in OC, with low genetic concordance between the primary-recurrent OC, and hinting that they are unlikely to be conventional drivers of OC.

These findings we have described above based on data from the GCP of FoundationOne^®^CDx (F1CDx) panel in the 20 pairs, which has limitations in genome coverage and sample size. Therefore, further studies need more complete and comprehensive coverage of genome to confirm the molecular distinctiveness between the primary and recurrent OC and uncover the hallmark along OC development.

### Electronic supplementary material

Below is the link to the electronic supplementary material.

Supplementary Material 1

Supplementary Material 2

Supplementary Material 3

Supplementary Material 4

Supplementary Material 5

Supplementary Material 6

Supplementary Material 7

## Data Availability

No datasets were generated or analysed during the current study.
