# Supplementary figures and images for: Genomic alteration discordance in the paired primary-recurrent ovarian cancers: based on the comprehensive genomic profiling (CGP) analysis
Source: J Ovarian Res. 2024 Jun 27;17:133. doi: 10.1186/s13048-024-01455-8 (PMC11212203; doi:10.1186/s13048-024-01455-8)

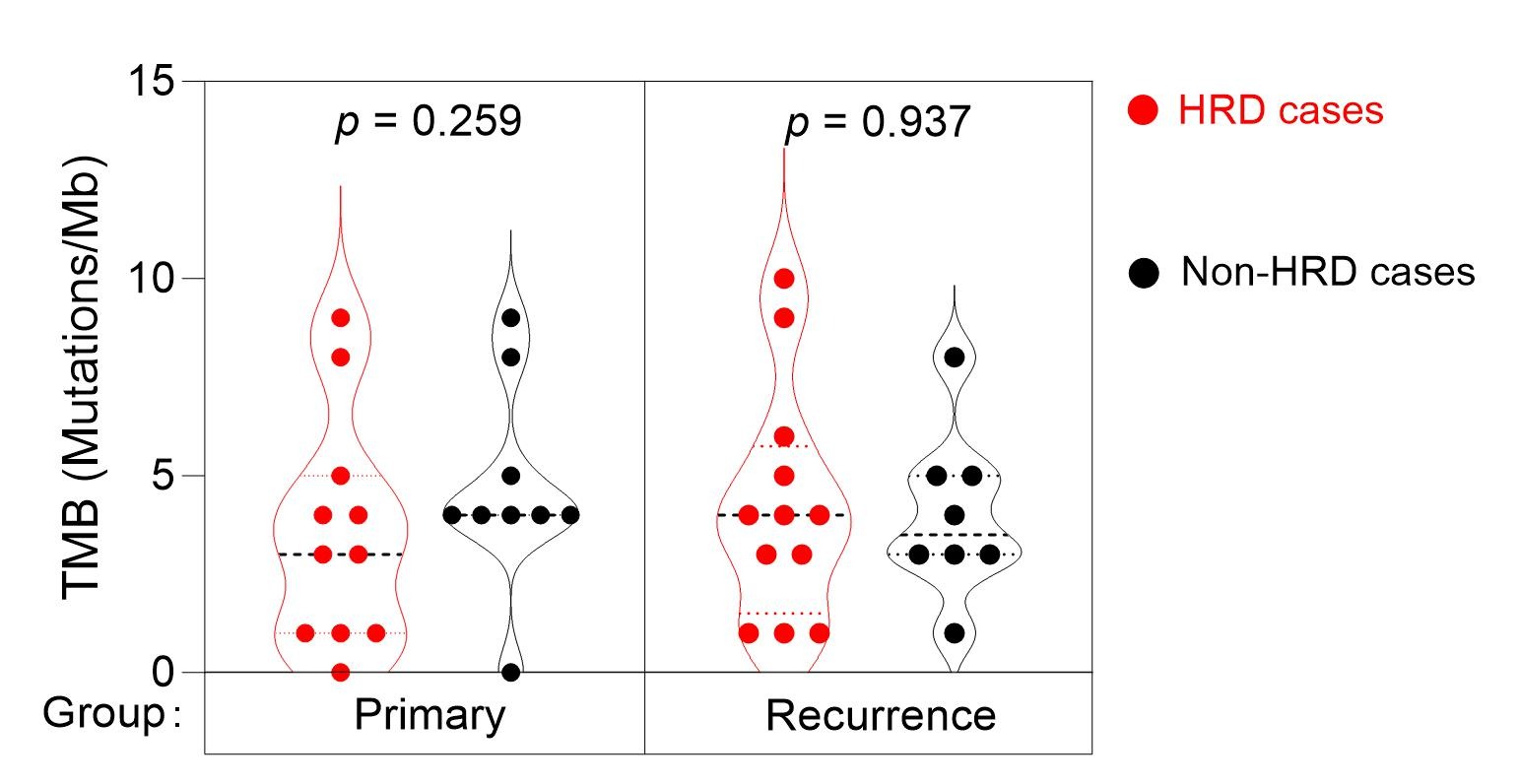

Supplement: Supplementary file 2 — Supplementary Material 2 [file 13048_2024_1455_MOESM2_ESM.jpeg]

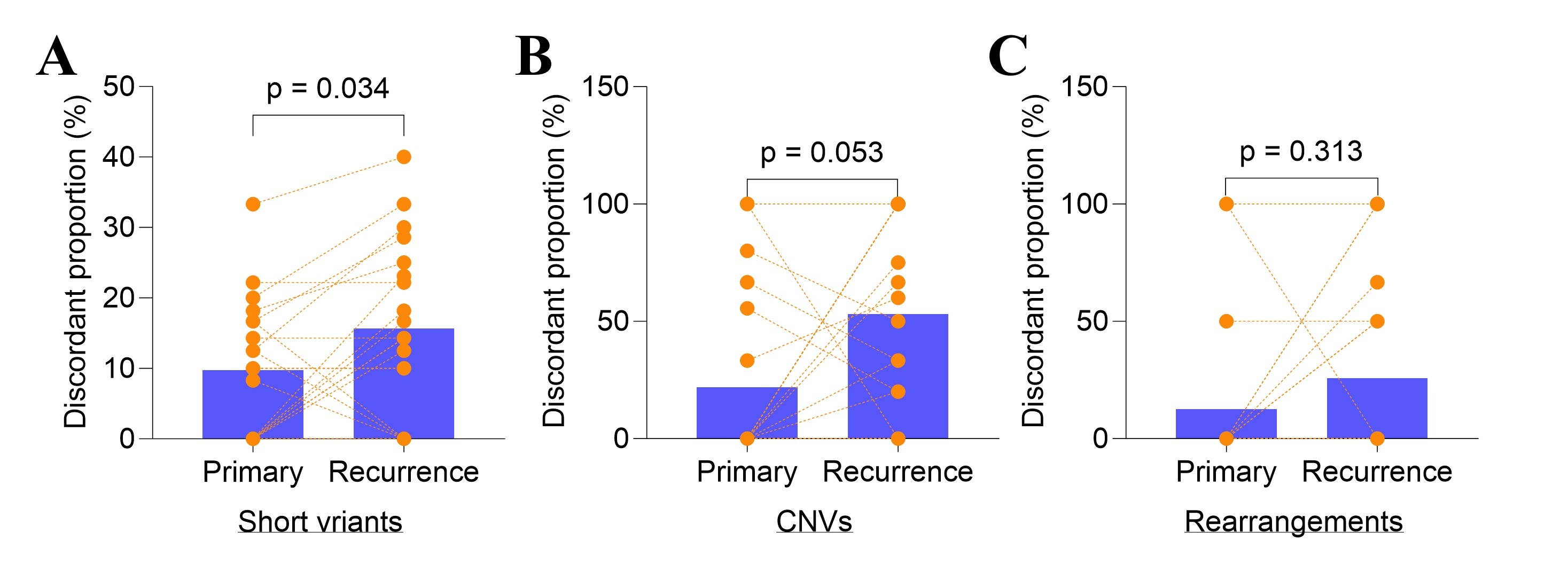

Supplement: Supplementary file 3 — Supplementary Material 3 [file 13048_2024_1455_MOESM3_ESM.jpeg]

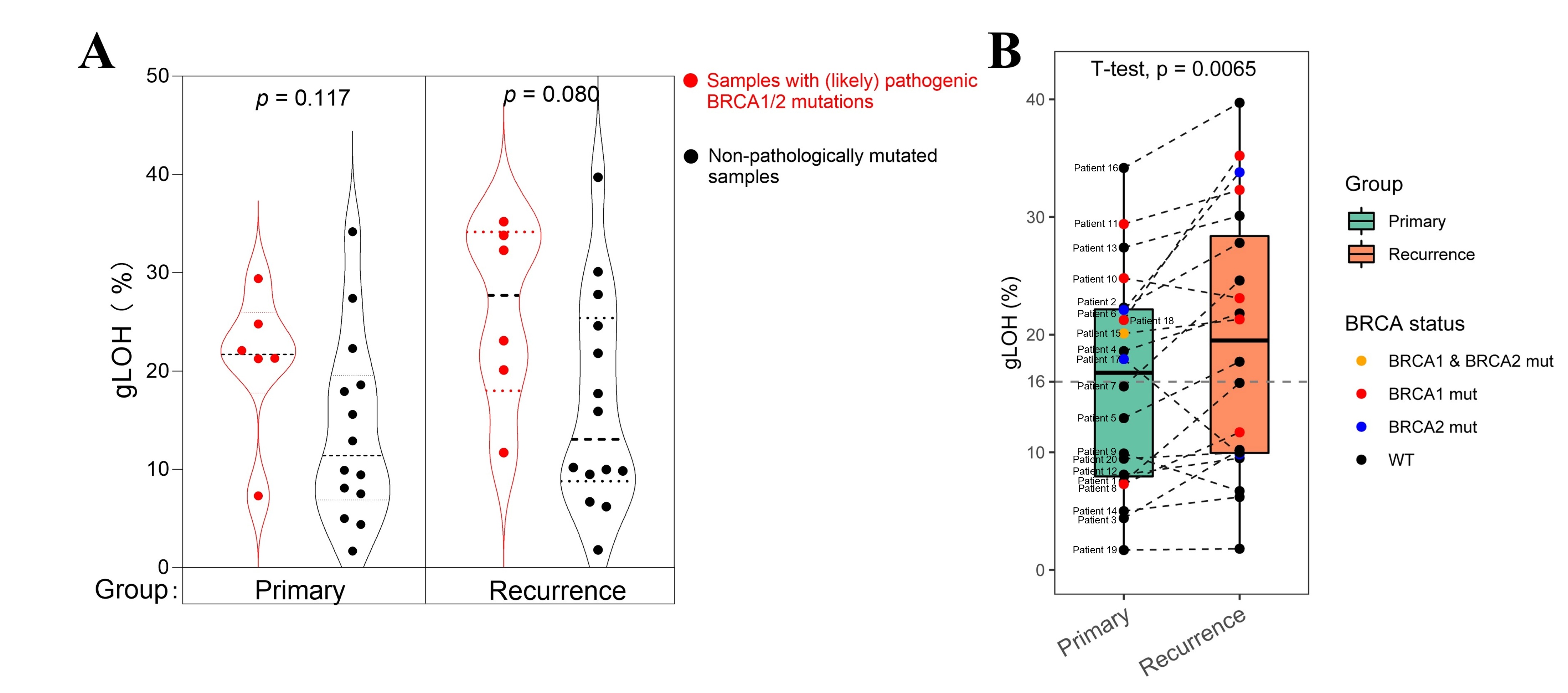

Supplement: Supplementary file 4 — Supplementary Material 4 [file 13048_2024_1455_MOESM4_ESM.jpeg]

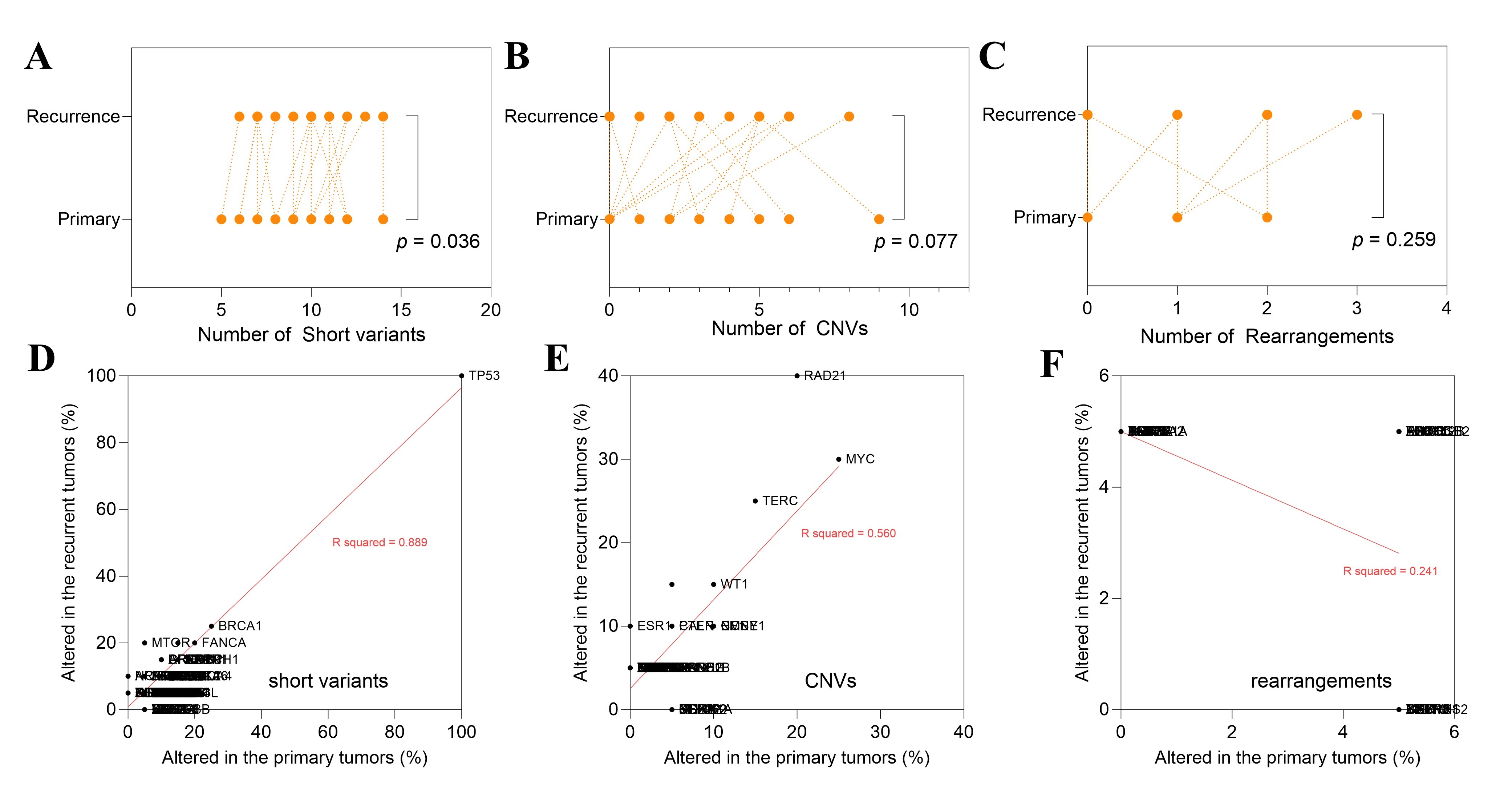

Supplement: Supplementary file 5 — Supplementary Material 5 [file 13048_2024_1455_MOESM5_ESM.jpeg]

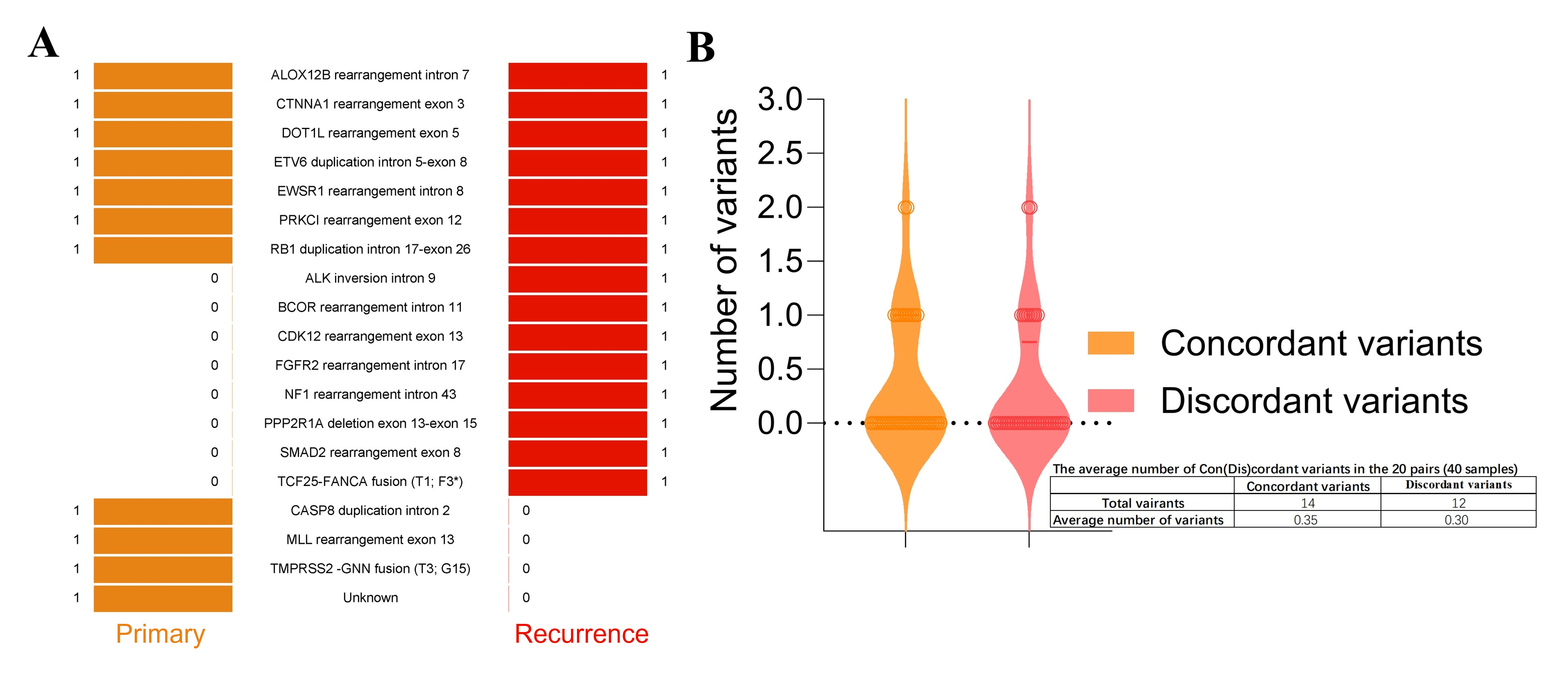

Supplement: Supplementary file 6 — Supplementary Material 6 [file 13048_2024_1455_MOESM6_ESM.png]
